# HD mouse models reveal clear deficits in learning to perform a simple instrumental response

**DOI:** 10.1371/currents.RRN1282

**Published:** 2011-11-30

**Authors:** Stephen Oakeshott, Russell G Port, Jane Cummins-Sutphen, Judy Watson-Johnson, Sylvie Ramboz, Larry Park, David Howland, Dani Brunner

**Affiliations:** ^*^Principal Scientist at PsychoGenics, Inc.; ^†^Research Associate at PsychoGenics Inc; ^§^Breeding Manager at PsychoGenics, Inc.; ^¶^VP, Neurodegenerative Disorders at PsychoGenics, Inc; ^#^Director, PreClinical Research, CHDI Foundation, Inc; ^**^Director of In Vivo Biology, CHDI Foundation Inc and ^††^Senior VP Behavioral R&D at PsychoGenics, Inc.

## Abstract

Mouse models of Huntington’s disease (HD) were trained to acquire one of two simple instrumental responses (a lever press or a nosepoke) to obtain food reinforcement. Animals from several HD strains revealed apparently progressive deficits in this task, being significantly less able than littermate controls to perform the required responses, at ages where motor function is only mildly affected. These data could provide a simple way to measure learning deficits in these mouse models, likely related to the characteristic pattern of neural damage observed in HD mouse models.

## ***Introduction***

Instrumental learning is thought to be dependent on two related processes or systems; a stimulus-response habit mechanism and a goal-directed process that might be described as response-outcome learning [1]. While there is a wide literature describing the specific and separate neural substrates of these processes (see e.g. [2]), the frontal-striatal areas known to be affected by HD in both human patients [3] and in many mouse models of the disease [4] are clearly implicated in this learning, which might therefore be expected to be impaired in HD mouse models.    

Accordingly, a series of experiments was carried out on several different lines of HD mice to test their ability to acquire a simple instrumental response in order to gain access to food. Animals were tested at ages where their motor capacities were relatively intact, such that any deficits observed could not simply be attributed to physical difficulties with performing the response. The initial studies were carried out with the R6/2 mouse [5], a widely studied transgenic fragment model that is known to reveal both cognitive [6] and motor [7] [8]deficits from an early age. Previous testing in a timing task (see [9]) has revealed that these R6/2 mice are able to acquire a lever press response at a very early age (4 weeks), so the focus of these initial studies was on slightly older animals, with testing from 7 and nearly 9 weeks of age.

## ***Experiment 1: Lever press acquisition in an R6/2 mouse carrying ~120 CAG repeats (R62 CHDI-001-4).***

### *Subjects*

R6/2 mice carrying ~120-CAG repeats on a mixed CBAJ/C57Bl6J background strain background, along with littermate controls, were bred in our facility by crossing ovarian transplant female animals with WT males. After weaning, animals were single housed in standard mouse cages and maintained throughout on a standard 12:12 light cycle with free access to water, with all testing conducted during the light period of the cycle. Subjects were maintained at 85% of their ad libitum body weight, factoring in expected growth, by daily feeding of limited quantities of food (BIO-SERV 500mg pellets). Two separate groups of female animals were trained, with the mice aged 7 weeks ± 3 days and 8½ weeks ± 2 days on the initial day of lever press training.   

These studies were carried out in strict accordance with the recommendations in the Guide for the Care and Use of Laboratory Animals, NRC 1996.  The protocol was approved by the Institutional Animal Care and Use Committee of Psychogenics, Inc. (PHS OLAW animal welfare assurance number A4471-01), an AAALAC International accredited institution (Unit #001213).

### *Equipment*

Mice were tested in standard mouse operant chambers (Med Associates, VT), each containing two retractable levers on either side of a food magazine. A nosepoke recess was also available on the opposite wall to the food magazine, which could be illuminated by a small embedded bulb. The chambers were located within individual sound attenuating shells, with a fan mounted at one end of the sound-attenuating cubicle that was active throughout. Reinforcement was provided by time-limited access to a dipper containing evaporated milk (Carnation™) delivered via a dipper. The hardware was controlled and all events were recorded by the Med-PC IV software package.

### *Behavioral procedures*

Following food restriction and magazine training, all animals were trained to lever press via a simple free operant procedure where a single lever was inserted throughout a 40 min session and lever pressing was reinforced with 4-s access to an evaporated milk reinforcer on a response-initiated FI20 schedule. No reinforcement was delivered without a lever press. Animals were trained to a criterion, requiring them to obtain 50 reinforcers across 2 consecutive sessions. Training was carried out daily Monday to Friday, with the animals resting over the weekends and continued for 12 sessions.

### *Results*

The proportion of mice to reach the acquisition criterion in these experiments was assessed using Kaplan-Meier event analysis over the entire acquisition test period, with an alpha level of 0.05 adopted throughout.   

R6/2 transgenic mice showed a marked delay in acquisition to criterion (Figure 1), with a high proportion of animals failing to acquire the response within the period tested. Analysis of these data through Kaplan-Meier survival analysis confirmed that the genotype effects were significant at both 7 weeks, Logrank (Mantel-Cox)  *χ*²(*1*) =  4.04, * p < 0.05*, and 9 weeks, Logrank (Mantel-Cox)  *χ*²(*1*) =  13.6, *p < 0.01*.  

**Figure:**
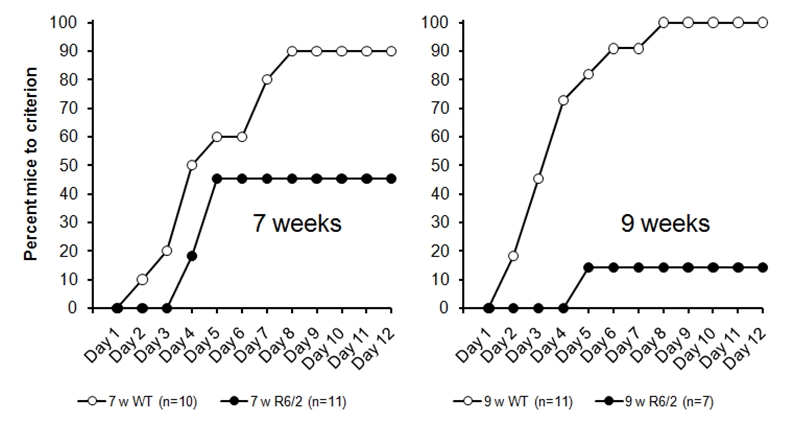
*Figure 1: Acquisition performance in the R62 (CHDI-001-4) mouse line, with testing from 7 weeks (left panel) and 9 weeks (right panel) in separate cohorts of mice.*

## ***Experiment 2: Lever press acquisition in an R6/2 mouse carrying ~240 CAG repeats (R62 CHDI-004-1).***

Although the age at which we saw deficits is very early, we considered the possibility that motor deficits are contributing to the observed phenotype as this line of R6/2 mice are known for the severity and early onset of their disease-related decline. In order to investigate more fully the potentially progressive nature of these deficits, a further evaluation was conducted of a slightly different R6/2 line, this one carrying a significantly expanded CAG repeat length (~240 repeats, up from ~120 in the previous study), which are known to have a delayed onset of the HD phenotype (see [8]).

### *Subjects*

R6/2 mice, along with littermate controls, were generated in house at Psychogenics by crossing hemizygous R6/2 males with WT females, all on a congenic C57Bl6J background. Animals were housed and maintained as in Experiment 1.   

Three separate groups of male animals were trained, with the mice aged 5 weeks ± 2 days, 7 weeks ± 3 days and 11 weeks ± 5 days on the initial day of lever-press training.

### *Equipment and procedures*

All equipment and procedures were as described in Experiment 1, except that only 10 training sessions were carried out.

### *Results*

There was a progressive deficit in these animals (Figure 2) but one that appears less severe than that seen in the R6/2 mixed background animals with ~120-CAG repeats evaluated in Experiment 1. In these data, while there is impaired response acquisition at 11 weeks (right panel) there do not appear to be significant deficits at 5 weeks (left panel) or 7 weeks (middle panel). Analysis of these data via Kaplan-Maier survival analysis confirmed the presence of a significant impairment at 11 weeks, Logrank (Mantel-Cox) *χ*²(*1*) = 8.34, *p < 0.01*, but no significant differences at 5 weeks, Logrank (Mantel-Cox)  *χ*²(*1*) = 0.03, * p > 0.8*, or 7 weeks Logrank (Mantel-Cox)  *χ*²(*1*) =  1.63, *p > 0.2*.

**Figure:**
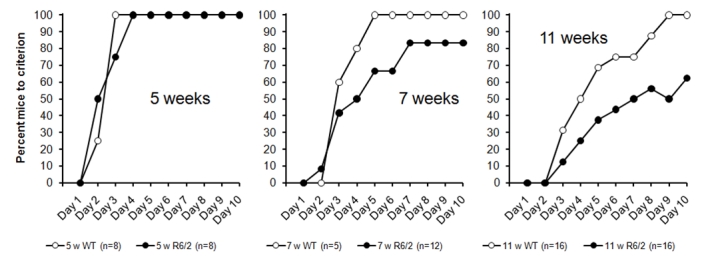
*Figure 2: Acquisition performance in the R62 (CHDI-004-1) mouse line, with testing from 5 weeks (left panel), 7 weeks (central panel) and 11 weeks (right panel) in separate cohorts of mice.*

## ***Experiment 3: Nosepoke acquisition in an R6/2 mouse carrying ~240 CAG repeats (R62 CHDI-004-(1)(4)).***

A further experiment was carried out to investigate if these findings might also be present in animals on a slightly different background, using a simple nosepoke response instead of a lever press. While it appears that these deficits are not simply related to motor problems in these R6/2 animals, this experiment aimed to confirm that a similar pattern of results might be detected in learning to perform a slightly different operant response, a nosepoke. 

### *Subjects*

R6/2 mice carrying ~240-CAG repeats on a C57Bl6J x CBAJ F1 background, along with littermate controls, were bred in our facility by crossing ovarian transplant C57Bl6J female animals with WT CBAJ males. In this study, animals were weaned into paired housing conditions in opti-MICE cages (Animal Care Systems, CO), remaining in these groupings throughout the experiments, with all other conditions matched to Experiment 1.    

Two mixed-sex cohorts of 8 mice per sex per genotype were tested, the initial cohort aged 6 ½ weeks ± 1 day at the start of testing, while the second cohort were all aged  7 weeks exactly on the first day of testing. One R6/2 female animal in the second cohort died of unrelated causes during the course of testing, so no data from this animal was included. 

### *Behavioral procedures*

Following food restriction and magazine training, all animals were trained to nosepoke via a simple free operant procedure, where nosepoking was reinforced throughout a 40 min test session with 4-s access to an evaporated milk reinforcer on a response-initiated FI20 schedule. No reinforcement was delivered without a nosepoke. Animals were trained to a criterion, requiring them to obtain 50 reinforcers across 2 consecutive sessions, with training carried out daily Monday to Friday and the animals resting over the weekends. Training continued for 20 sessions.

### *Results*

This experiment confirmed that the R6/2 deficit observed in Experiments 1 and 2 is generalized to the acquisition of a nosepoke response and to animals on this CBAJ x C57Bl6J F1 background (Figure 3). In both cohorts, a clear deficit is visible in the R6/2 mice, which appears slightly more pronounced in the 7 week animals (right panel). Statistical evaluation of these cohorts confirmed that this learning proceeded less readily in the R6/2 mice than in WT controls, with significantly reduced acquisition in both cohorts, Logrank (Mantel-Cox)  *χ*²(*1*) = 4.85 and Logrank (Mantel-Cox)  *χ*²(*1*) = 12.7, all * ps < 0.03.*

**Figure:**
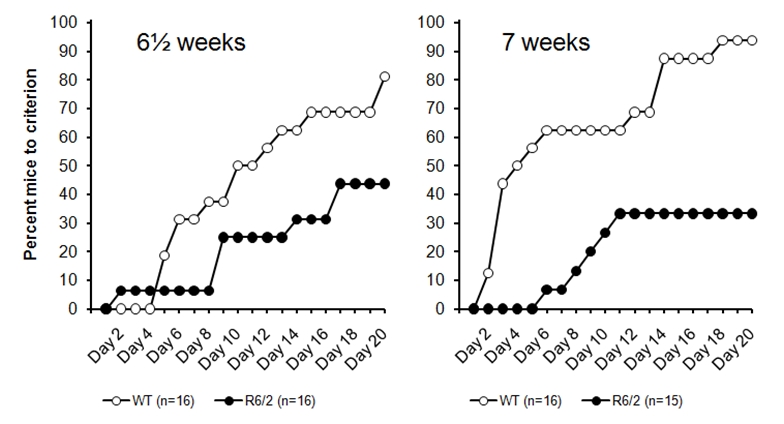
*Figure 3: Nosepoke acquisition performance in the R62 (CHDI-004-(1)(4)) mouse line, with testing from 6 ½ weeks (left panel) and  7 weeks (right panel) in separate cohorts of mice.*

## ***Experiment 4: Lever press acquisition in the BAC HD (CHDI-007-(3)(1)) mouse.***

To determine whether this deficit might generalize to a rather different model of Huntington’s disease, a small cohort of BAC HD mice [10], carrying a full-length mutant human gene, were also tested, with these mice at a much more advanced age than the previously tested R6/2 animals in order to allow a potential phenotype to develop.

### *Subjects*

BAC HD mice, carrying ~97 stable CAGCAA repeats on a C57Bl6J x FVB/NJ F1 background were bred at the Jackson Laboratory (Bar Harbor, ME) and shipped to the test facility as adults. Mice were maintained throughout in opti-MICE cages (Animal Care Systems, CO) on a 12:12 light cycle with free access to water. Once acclimated to our colony, the animals were pair-housed and food restricted, with WT mice reduced to 85% of their free-feeding body weights.    

The BAC mice, which develop excess fat deposits, were restricted gradually to the point where their food consumption matched that of the 85% WT animals in a 30-min free feeding test, at which point they were slightly heavier than controls (mean 32.8g for BAC mice, 27.4g for WT controls at the start of training). A single group of BAC HD mice was evaluated, with the animals at 74 weeks of age ± 1 week at the start of lever training.

### *Behavioral procedures*

Following food restriction, exactly the same training procedure was employed as in Experiments 1-3.

### *Results*

No differences were recorded in lever press acquisition in these animals (Figure 4), with all mice rapidly acquiring the lever press response, an impression confirmed by Kaplan-Maier analysis, Logrank (Mantel-Cox)  *χ*²(*1*) = 1.19, *p > 0.25.*

**Figure:**
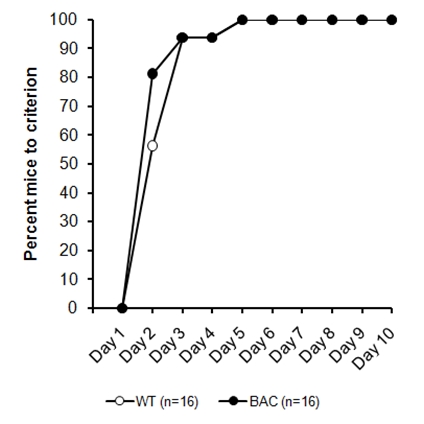
*Figure 4: Lever press acquisition performance in the BAC HD (CHDI-007-(3)(1)) mouse line, with testing from 74 weeks.*

## ***Experiment 5: Nosepoke acquisition in the z_Q175 KI (CHDI-015-1) mouse.***

Finally, a novel knock-in model generated at Psychogenics (see [11] for detailed description of this line), the z_Q175 KI mouse, derived from the CAG 140 mouse [12], was evaluated, to confirm that the phenotype observed is not specific to the R6/2 mouse. 

### *Subjects*

Homozygous, heterozygous and wild type knock-in mice were bred in our facility by crossing pairs of z_Q175 heterozygous mice, all on a congenic C57Bl6J background. Prior to this testing, these animals underwent testing in a comprehensive behavioral battery, involving a variety of motor and cognitive tests, but were naïve to operant boxes and had never before been food restricted.  The mice were maintained throughout in opti-MICE cages (Animal Care Systems, CO) on a 12:12 light cycle with free access to water.   

Two cohorts of animals were tested; a small cohort of male mice, evaluated from the age of 74 weeks, and a larger mixed sex cohort evaluated from the age of 53 weeks.

### *Behavioral procedures*

Test procedures were exactly as in Experiment 3 above.

### *Results*

As was the case with the R6/2 animals described above, there are clear deficits in acquisition of a nosepoke response in the z_Q175 homozygous mice at both 74 and 53 weeks, with the phenotype appearing more advanced in the older mice (Figure 5). Kaplan-Meier analysis was not possible in the 74 week group since no z_Q175 homozygous animals acquired the response within the available timeframe (also note the small n in this cohort) but analysis of the 53 week animals confirmed that there were highly significant differences amongst the groups, Logrank (Mantel-Cox)  *χ*²(*2*) = 32.8, *p < 0.0001*.

**Figure:**
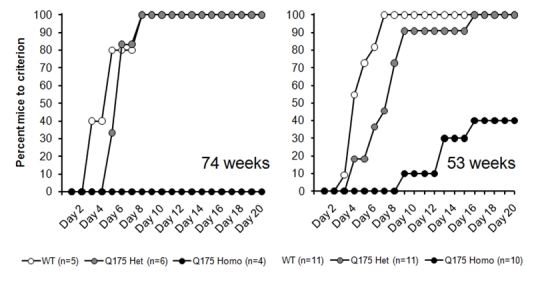
*Figure 5: Nosepoke acquisition performance in the z_Q175 KI (CHDI-015-1) mouse line, with testing from 74 weeks (left panel) and 53 weeks (right panel) in separate cohorts of mice.*

## ***Discussion***

This series of simple experiments clearly indicates that marked deficits in instrumental learning can be readily measured in the HD mouse models studied. In combination, the implication of these data is that an inability to acquire an operant response is a fundamental feature of the R6/2 phenotype, with similar deficits visible in two rather different strains of R6/2 animals, with two quite dissimilar instrumental responses (a lever press and a nosepoke) and irrespective of the sex of the animals. Similar deficits are seen in nosepoke acquisition in a novel KI line with a long CAG repeat (the z_Q175 KI model, see [11]), though not in a full-length transgenic model, the BAC HD mouse (see [11]). 

While the affected animals are known to have motor deficits, these should not be severe enough at the ages tested to significantly impair performance of these simple responses [8] [11]; indeed, in separate studies, R6/2 mice in our hands continue performing a previously learnt lever-press response at an age where clear and severe deficits are observed in novel response acquisition, while animals quite unable to acquire a nosepoke response in these experiments continue to successfully retrieve food from the magazine, requiring an identical level of physical exertion.   

The exact nature of these deficits will require further work to define the relative contributions of impaired learning, memory, or reduced motivation to work for food, but similar deficits have been reported even in more subtly impaired mouse lines. A recent study in the Hdh(Q92/Q92) knock-in mouse model revealed early deficits in learning to perform a more complicated motor task, nosepoking in a series of lit recesses [13].    

More generally, operant experiments can be an ideal tool for evaluation of the cognitive phenotype in mouse disease models (see [14] for an overview), but one concern with more rapidly progressing models like the R6/2 is that many operant learning paradigms can require several weeks of training, such that the compressed lifespan of these models may preclude their use. Given that the current experiments reveal such clear deficits in a very quick training procedure, however, this type of simple response learning paradigm might be ideal for initial screening of such models, albeit with the caveat that the deficits revealed may not be specific to any one region of particular interest.

## ***Acknowledgements***

Thanks to Simon Brooks, Stephen Dunnett, Bernard Balleine, Liliana Menalled, Carol Murphy and Fuat Balci for helpful discussion and to Melinda Ruiz, David Connor and Diana Carlsen for technical support and assistance.

## ***Funding Statement***

## CHDI Foundation is a not-for-profit biomedical research organization exclusively dedicated to discovering and developing therapeutics that slow the progression of Huntington’s disease. The research described in this manuscript was funded by CHDI Foundation, conceptualized and planned by all authors listed, and conducted at PsychoGenics, Inc.  

## ***Competing Interests Statement***

Stephen Oakeshott, Russell Port, Judy Watson-Johnson, Sylvie Ramboz and Dani Brunner are or were all employed by PsychoGenics, Inc., a for-profit institution.    

CHDI Foundation provides financial support to *PLoS Currents: Huntington Disease*. Editorial responsibility for the content in *PLoS Currents: Huntington Disease* rests entirely with PLoS, the Editors, and Board of Reviewers.   

The authors have declared that no further competing interests exist.
